# Epstein–Barr virus‐positive mucocutaneous ulcer: A unique case occurring in association with cholelithiasis in a gallbladder

**DOI:** 10.1002/jgh3.12425

**Published:** 2020-10-09

**Authors:** Mohmmed Awadh, John O'Mahony, Ciara Ryan, Fiona Quinn, Larry Bacon, Narayanasamy Ravi, Richard Flavin

**Affiliations:** ^1^ Department of Histopathology St. James's Hospital and Trinity College Dublin Dublin Ireland; ^2^ Department of Radiology St. James's Hospital and Trinity College Dublin Dublin Ireland; ^3^ Cancer Molecular Diagnostics St. James's Hospital and Trinity College Dublin Dublin Ireland; ^4^ Department of Haematology St. James's Hospital and Trinity College Dublin Dublin Ireland; ^5^ Department of Surgery St. James's Hospital and Trinity College Dublin Dublin Ireland

**Keywords:** Cholelithiasis, Epstein‐Barr Virus, EBVMCU, Gallbladder

## Abstract

Epstein–Barr virus (EBV)‐positive mucocutaneous ulcer is a lymphoproliferative disorder occurring in patients due to iatrogenic or age‐related immunosuppression confined to the oropharynx, skin, and gastrointestinal tract. Here, we report the first case to our knowledge of EBV‐positive mucocutaneous ulcer occurring in a gallbladder.

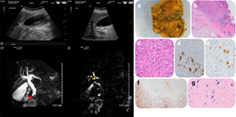

An Epstein–Barr virus (EBV)‐positive mucocutaneous ulcer (EBVMCU) is a lymphoproliferative disorder occurring in patients due to iatrogenic or age‐related immunosuppression^1^ and was first described as a distinct clinicopathological entity in 2010 by Dojcinov *et al*., who reported 26 patients with ulcerative lesions confined to the oropharynx, skin, and gastrointestinal tract.^2^ The disease generally has a self‐limited course with spontaneous regression in some cases.

An 82‐year‐old female presented with a 4‐day history of painless jaundice preceded by general malaise and bilious vomiting. She had a past medical history of ischemic heart disease, osteoarthritis, gastritis, duodenitis, and postherpetic neuralgia. She was on multiple medications, primarily for ischemic heart disease, along with supplementary multivitamins. General investigations revealed obstructive liver function test (LFT)s.

An ultrasound demonstrated mobile sludge within the gallbladder but no wall thickening or pericholecystic free fluid. The common bile duct (CBD) was noted to be dilated (measuring up to 14 mm), with associated intrahepatic duct dilatation. No pancreatic or liver parenchymal abnormalities were noted (Fig. 1).

**Figure 1:**
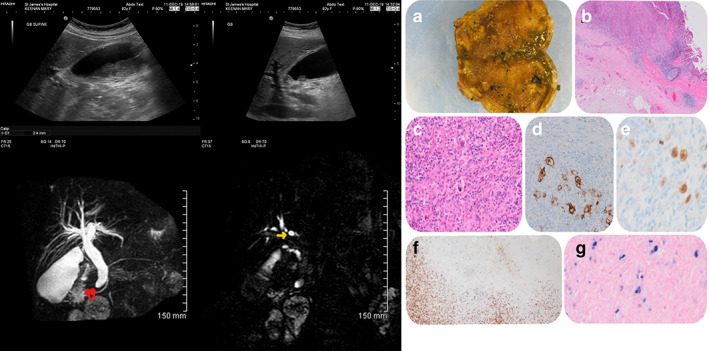
Abdominal ultrasound (left top) and magnetic resonance cholangiopancreatography (left bottom); (a) gross macroscopy, note the white nodular area around gallbladder neck; (b) low‐power view (hematoxylin eosin, 4×); (c) high‐power view (hematoxylin eosin, 40×); (d) CD30; (e) PAX‐5; (f) CD3; (g) EBER.

Subsequent magnetic resonance cholangiopancreatography (MRCP) confirmed CBD and intrahepatic duct dilatation. Gallbladder sludge was identified, in addition to cholelithiasis. A filling defect was identified in the proximal cystic duct (Fig. 1 yellow arrow), likely representing a calculus. While no intraluminal filling defect was identified in the CBD, abrupt tapering of the distal CBD, suggestive of a stricture, was noted (Fig. 1 red arrows). An endoscopic ultrasound was recommended for further evaluation. Iatrogenic perforation of the duodenum at endoscopic retrograde cholangiopancreatography (ERCP) led to laparotomy, repair, and cholecystectomy.

Macroscopically, the gallbladder measured 6 5 × 55 × 25 mm, and on sectioning, there was a 25‐mm firm, white nodule proximally (Fig. 1a); the remaining gallbladder mucosa was stippled brown with multiple small black gallstones stones 1–4 mm in size. Microscopically, the nodular area in the gallbladder showed discrete circumscribed areas of ulceration, with an underlying polymorphic population of small lymphocytes, prominent histiocytes, plasma cells, and eosinophils together with scattered Hodgkin Reed Sternberg‐like cells and a base of small lymphocytes (Fig. 1b,c). Background gallbladder mucosa showed evidence of acute‐on‐chronic (follicular) cholecystitis. On immunohistochemistry, the Hodgkin‐like cells were positive for CD30, CD20, CD79a, PAX‐5, OCT‐2, BOB‐1, BCL6, BCL2, and MUM‐1 and negative for ALK‐1 and CD10 (Fig.1d,e). There were CD3‐positive T cells at the base of the lesion (Fig. 1f). EBV in situ hybridization (ISH) stained a range of cell sizes, including the Hodgkin‐like cells and background smaller lymphocytes, including within areas of follicular cholecystitis (Fig. 1g). Multiplex polymerase chain reaction (PCR) detected clonal immunoglobulin heavy‐chain (VFR3‐J) and weak clonal immunoglobulin light‐chain kappa (V‐J) gene rearrangements in the patient's sample. The appearances were consistent with an EBVMCU. On follow‐up radiological imaging, there was no lymphadenopathy. On hematology follow up 6 weeks later, she was well with no B symptoms.

Here, we describe the first case of EBVMCU arising within the gallbladder. Previous reports of an association between EBV and hepatobiliary pathology indicate that EBV‐related hepatitis is recognized as an important cause of cholestasis. In addition, isolated gallbladder wall thickness has been reported in patients with infectious mononucleosis, although acute acalculous cholecystitis (AAC) is an atypical presentation of primary EBV infection.^3^ AAC can be seen in any age group, but it is most commonly seen in the fourth or eighth decades of life. AAC represents 30–50% of all cases of acute cholecystitis presenting in the pediatric population.^4^

In contrast, EBVMCU typically arises in locations following local trauma or inflammation in immunosuppressed patients and is consistently associated with EBV.^5^ In this particular case, the patient had no history of immunodeficiency, nor were she on any immunosuppressant drugs. We speculate that, in this case, the EBVMCU is attributable to advanced age and local trauma associated with cholelithiasis and resultant cholecystitis and is a diagnosis to be aware of in elderly patients.
